# Silver Nanoparticles, Ions, and Shape Governing Soil Microbial Functional Diversity: Nano Shapes Micro

**DOI:** 10.3389/fmicb.2016.01123

**Published:** 2016-07-25

**Authors:** Yujia Zhai, Ellard R. Hunting, Marja Wouters, Willie J. G. M. Peijnenburg, Martina G. Vijver

**Affiliations:** ^1^Institute of Environmental Sciences, Leiden UniversityLeiden, Netherlands; ^2^National Institute of Public Health and the EnvironmentBilthoven, Netherlands

**Keywords:** nanosilver, Biolog Ecoplates, nanoecotoxicology, microbial community, functional diversity

## Abstract

Silver nanoparticles (AgNPs) affect microbial metabolic processes at single cell level or lab-culture strains. However, the impact of different AgNPs properties such as the particle, ion release, and shape on functional responses of natural soil microbial communities remain poorly understood. Therefore, we assessed the relative importance of particles and ions of AgNPs in bacterial toxicity and how the functional diversity of soil microbial communities were impacted by AgNPs shapes (i.e., plates, spheres, and rods) in laboratory incubations. Our results showed that the relative contribution of AgNPs_(particle)_ increased with increasing exposure concentrations (accounted for about 60–68% of the total toxicity at the highest exposure level). In addition, the functional composition of the microbial community differed significantly according to different AgNPs shapes. The various properties of AgNPs thus can significantly and differentially affect the functional composition of microbial communities and associated ecosystem processes depending on the level of environmental exposure.

## Introduction

Silver nanoparticles (AgNPs) are known for their anti-microbial properties and are broadly used in clothing, food industry, cosmetics, and medical devices (Kendra and Arturo, 2014; Theophel et al., 2014). Likely resulting from these industrial and medical applications, AgNPs are reported to end up in both soils and aquatic sediments (e.g., Schlich et al., 2013; Yu et al., 2013; McKee and Filser, 2016), with inherent risks for natural microbial communities that play a pivotal role in the functioning of ecosystems (Lau and Lennon, 2012).

While most studies have focused on AgNPs toxicity on single bacterial species under laboratory conditions (e.g., Pal et al., 2007; McQuillan et al., 2011; Levard et al., 2012), a number of studies also provided evidence that small size metal NPs are detrimental to natural soil microbial communities (Dinesh et al., 2012), affecting community composition (Kumar et al., 2011; Echavarri-Bravo et al., 2015; Sillen et al., 2015), bacterial biomass (Hänsch and Emmerling, 2010), and bacterial processes (Ge et al., 2011). Toxicity of AgNPs suspensions, however, can be ascribed to the properties of the nanoparticles and/or to dissolved ions released from the AgNPs. Which property dominates toxicity appears contradictive as exposure of various bacterial and fungal species to both particle species and ion species of AgNPs has to date revealed that both species can be very important (cf. Levard et al., 2013; González et al., 2015; Tlili et al., 2016). Moreover, most studies evaluating the relative importance of particle species and ion species of AgNPs were restricted to single bacterial species/strains, and therefore, the relative contributions of particle species and ion species of AgNPs to natural microbial communities remain uncertain.

In addition to ion and particle effects, the particle attributes size and in particular shape have been well documented to determine toxicity of NPs in laboratory cultures. Ivask et al. (2014) analyzed the toxicity of different sized AgNPs on a variety of species showed that responses were often size-dependent, having the highest impact with small particles. Additionally, the shape showed to have an impact on responses, e.g., George et al. (2012) reported that silver nanoplates were considerably more toxic than spheres and wires. Moreover, silver nanocubes displayed a lower toxicity compared to quasi-spherical silver nanoparticles and silver nanowires (Gorka et al., 2015). However, a comprehensive understanding on the effect of AgNPs shape on natural microbial communities is currently lacking. Therefore, whether AgNPs shape governs microbial functional diversity on the community level requires investigation.

The present study aims to evaluate: (i) the relative contribution of particle species and ion species of AgNPs to the overall toxicity on microbial carbon substrate utilization as a relevant functional endpoint and (ii) the impact of AgNPs shape on the functional diversity of soil microbial communities. To this end, natural soil microbial communities were exposed to different concentrations and shapes (spheres, plates, and rods) of AgNPs in laboratory incubations.

## Materials and Methods

### Nanoparticles and Reagents

AgNP 50–80 nm nanoplates (stored as 450 mg/L Ag dispersions in polyvinylpyrrolidone) were obtained from Moscow State University (Moscow, Russia). A dispersion of AgNP 15 nm nanospheres [particle size 15 nm, 10.16% Ag (w/w) in a stabilizing vehicle material consisting of polyoxyethylene glycerol trioleate (4%; w/w) and polyoxyethylene (20) sorbitan mono-laurate (Tween 20; 4%; w/w; manufacturer’s information) (Van der Ploeg et al., 2014)] was obtained from a commercial source (Nanostructured & Amorphous Materials, Houston, TX, USA); AgNP 20–40 nm nanospheres (particle size range 20–40 nm, powder) were manufactured by a patented vapor condensation process from QSI-Nano^®^ (Santa Ana, CA, USA); AgNP 50 nm nanorods (with reported width of 50 nm and length of 5–10 μm, dispersion of 8.6% Ag (w/w) in an aqueous solution containing polyvinylpyrrolidone (<1% w/w), acrylic/acrylate copolymer (<2% w/w) and polycarboxylate ether (<2% w/w)) (Hund-Rinke and Schlich, 2014) were supplied by the Fraunhofer Institute (Schmallenberg, Germany). AgNO_3_ was purchased from Sigma–Aldrich (Zwijndrecht, Netherlands).

Physio-chemical characterizations of AgNPs were done following the risk assessment of engineered nanoparticles (ENPRA) nanomaterial dispersion protocol for toxicological studies (Kermanizadeh et al., 2012). The stock suspension was sonicated for 8 min (in 4°C water, twice) at 38 ± 10 KHz, and diluted in BIS–TRIS buffer. During this dilution process the primary AgNPs dispersions were continuously inverted and vortexed to prevent the particles from sedimentation. Characterization of particle size and morphology of the four kinds of AgNPs were analyzed using transmission electron microscopy (TEM; JEOL 1010, IEOL Ltd., Japan). Size distribution of AgNPs suspensions were analyzed at 1, 24, and 96 h after incubation in the exposure medium by dynamic light scattering (DLS, Malvern, Instruments Ltd., UK). Concentrations of AgNPs suspensions (designated as AgNPs_(total)_ hereafter) in the exposure media were determined using Atomic Absorption Spectroscopy (AAS; Perkin Elmer 1100B) at 1, 24, 48, 72, and 96 h. The Ag-ion concentrations released from the AgNPs_(total)_ at 1 mg/L in test media were also measured at the same times.

### Natural Soils and Microbial Community

Soil samples were collected from a site dominated by deciduous trees (52°07’06.7′′N 5°11′23.1′′E, Bilthoven, Netherlands). Moist soil samples were selected and sieved with an 8-mm sieve and kept at 4°C for the duration of the experiments. The soil moisture content was maintained at the initial content which was 18.4% of the dry soil weight by regularly supplying distilled water. Subsequently, 60 g soil subsamples were placed in glass jars and incubated for 120 h. Ten grams of this soil sample was added to 90 mL of 10 mM (2.09 g/L) BIS–TRIS (Sigma–Aldrich B9754). This buffer was adjusted to pH 7 using 1 M HNO_3_. The buffer with soil added was then centrifuged for 10 min at 1500 rpm, after which the supernatant was filtered (to remove the soil particles) and diluted five times with the same buffer (soil microbial community extract).

### Experimental Outline

A 15 mL soil extract of 1% w/v soil microbial community extract was added to the different silver suspensions treatments. Exposure concentrations were selected to range from 0.070 to 0.678 mg/L for the 50–80 nm nanoplates, 0.072 to 0.708 mg/L for the 15 nm nanospheres, from 0.108 to 0.814 mg/L for the 20–40 nm nanospheres and from 0.141 to 1.529 mg/L for the 50 nm nanorods based on range finding experiments. The effect of AgNO_3_ was tested in the range from 0.003 to 0.05 mg/L as a positive reference to assess the toxicity of Ag^+^. Considering the rapid aggregation process of nanoparticles, the soil microbial community extracts were exposed to AgNPs for less than 2 h. To obtain a solution of the ion species of AgNPs, the AgNPs suspensions were centrifuged at 30,392*g* for 30 min (Sorvall RC5Bplus centrifuge, Fiber lite F21-8 × 50 year rotor) and filtered by a filter with 0.02 μm pore diameter. This step yields supernatants with negligible amounts of particles (Hua et al., 2014), which were used as Ag-ion suspension. AgNP_(ion)_ was operationally defined as the Ag measured in the supernatant. Accordingly, AgNP_(particle)_ is the difference between the Ag measured in the AgNP suspension and AgNP_(ion)_.

Community metabolic diversity was determined by evaluating the carbon substrate utilization patterns using commercial Ecoplates (Biolog, Hayward, CA, USA; Garland and Mills, 1991) containing 3 times 31 carbon sources (Supplementary Table S1). Biolog Ecoplates are comprised of ecologically relevant, structurally diverse compounds, yet do not include e.g., recalcitrant substrates nor specific substrates typical of the soils used in this study. It is therefore impossible to directly relate substrate utilization profiles to the actual functioning of the soil microbial communities. Nonetheless, the number of substrates used can serve as a proxy of the metabolic diversity of the microbial community (Garland, 1999; Krause et al., 2014). Moreover, differences in utilization profiles indicate that functionally distinct microbial communities can develop depending on treatment (e.g., Hunting et al., 2013a,b, 2015; Wang et al., 2015). Each well of the Biolog Ecoplates was inoculated with 100 μl of supernatant extracted from different AgNPs exposed soil samples as described above, and incubated for 96 h at 20°C, to enhance microbial growth. Optical density (OD) was measured at 590 nm (OD_590_) immediately after inoculation of the plates and at 24, 48, 72, and 96 h to ensure that a saturation of the utilization rate in all samples was reached.

### Data Analysis

The average OD_590_ of each carbon source (three replicates) was calculated for each measurement. Average well color development (AWCD) was used to represent the average microbial metabolic activity. AWCD was calculated for the 31 substrates as described by (Garland and Mills, 1991) and transformed (Weber et al., 2007).

The transformed values of AWCD of soil extracts exposed to AgNPs were used as the total effect (*E*_(total)_). The effect of AgNO_3_ was also tested in order to allow to calculate the effect of ions released from the corresponding AgNPs (*E*_(ion)_). The response addition model (RA) was used to calculate the relative contribution to toxicity of AgNPs_(particle)_ and AgNPs_(ion)_ because different mechanistic pathways of NP_(particle)_ and NP_(ion)_ were assumed, according the assumptions of Hua et al. (2014):

$$\begin{array}{c} E_{\left(\text{total}\right)}\;=\;\left(\left(1-E_{\left(\text{ion}\right)}\right)\left(1-E_{\left(\text{particle}\right)}\right)\right) \end{array}$$

Substrate utilization was chosen as the transformed OD_590_ value greater than 0.25. Shannon indices (Shannon, 1948) were also calculated for each microbial community exposed to differently AgNPs as a measure of the diversity of the microbial communities active during incubation.

The exposure concentrations for each AgNPs are subject to exponential decay as they decrease along with time. This dissociation model heads downhill gradually reaching a plateau (*P*). The dynamics of the exposure concentrations were accounted using the time weighted average (TWA) concentrations for AgNPs_(total)_ and AgNPs_(particle)_ (1–96 h) depending on the nonlinear fit of ion release profiles:

$$\begin{array}{c} C\;=\;\left(C_{0}-P\right)\;*\;e^{\left(-K\;*\;T\right)}\;+\;P \end{array}$$

where *C*_0_ is the *C* (concentration, mg/L) value when *T* (time, h) is zero. *P* is the *C* value at infinite times. *K* is the rate constant. Statistics of the nonlinear fit of ion release profiles for each AgNPs_(total)_ and AgNPs_(particle)_ are shown in Supplementary Table S2.

The EC*_x_* values were based on TWA concentrations for AgNPs_(total)_ and AgNPs_(particle)_ and calculated by the dose–response-inhibition model in GraphPad Prism 6.0. Statistically significant differences between different soil extracts exposed to four AgNPs were determined by a two-way ANOVA and Turkey’s honestly significant difference tests (with significance level set as *p* < 0.05). To relate the microbial functional composition under four AgNPs exposure levels, the 31 carbon substrates utilization were analyzed using a Euclidean-based cluster analysis and a two-way analysis of similarities (ANOSIM) in PAST 3.0. (Hammer et al., 2001; Villéger et al., 2008).

## Results

### Physicochemical Characterization of AgNPs

**FIGURE 1:**
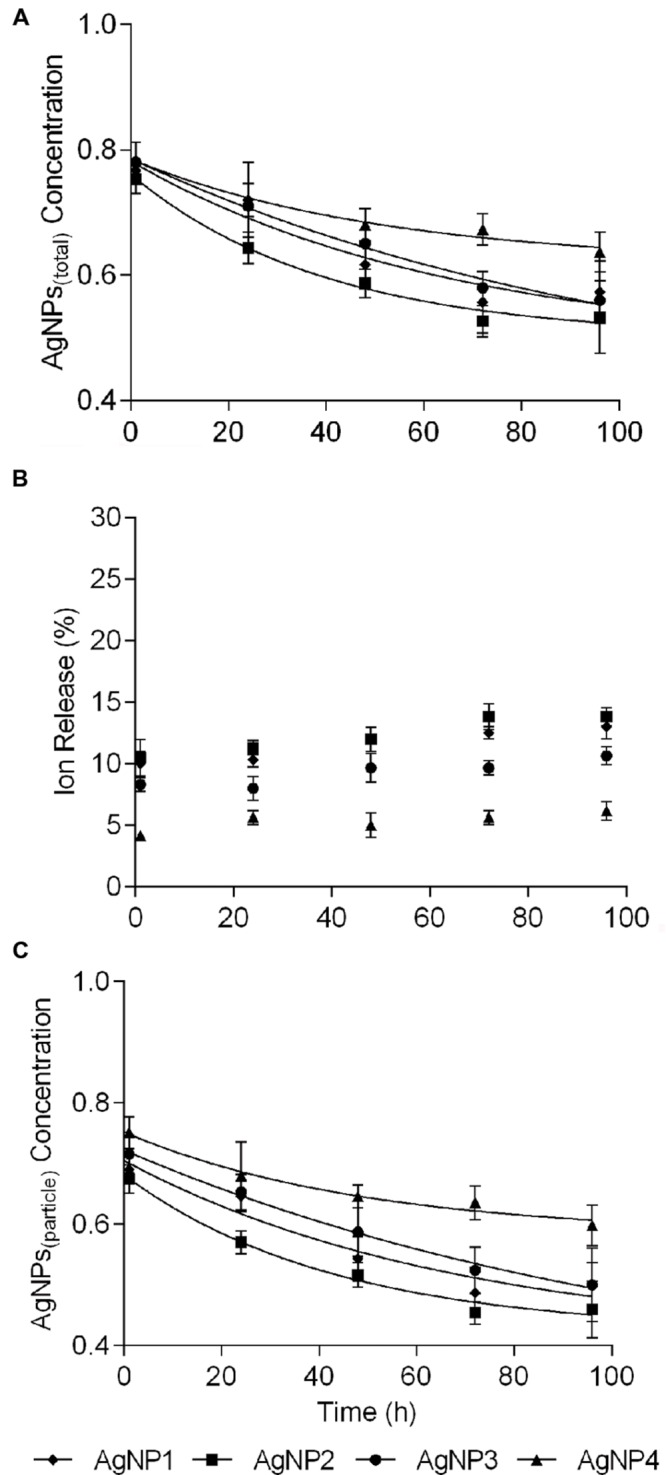
**Ion release profiles of silver nanoparticles (AgNPs) at 1 mg/L, during 96 h of incubation in the exposure medium. (A)** Total Ag concentrations of the AgNPs_(total)_. **(B)** Relative percentage of dissolved Ag released from AgNPs. **(C)** Ag concentrations of the AgNPs_(particle)_. Data are mean ± SD (*n* = 3). (AgNP1:50–80 nm nanoplates, AgNP2:15 nm nanospheres, AgNP3:20–40 nm nanospheres, and AgNP4:50 nm nanorods).

Transmission electron microscopy images reflecting the different shapes and primary sizes of the four AgNPs are shown in Supplementary Figure S2. Data on size distributions after 1, 24, 48, and 96 h are given in **Table 1** It can be seen that AgNPs aggregated directly after submerging into the exposure medium. The particle sizes in the exposure medium changed from 191, 196, 187, and 288 nm after 1 h of incubation to 368, 385, 373, and 496 nm after 96 h of incubation, respectively. The 96 h ion release profiles of AgNPs are shown in **Figure 1** It can be seen that the percentage of AgNP_(ion)_ in the AgNPs suspension at 1 mg shifted from 10, 8, 12, and 4% after 1 h to 13, 10, 14, and 6% after 96 h of incubation in the buffer, respectively.

**Table 1:** Hydrodynamic diameter of 1 mg/L suspensions of silver nanoparticles (AgNPs) in the exposure medium.

	Hydrodynamic diameter (nm)^a^			
Type	1 h	24 h	48 h	96 h
50–80 nm nanoplates	196 ± 15	352 ± 27	368 ± 41	382 ± 54
15 nm nanospheres	191 ± 12	346 ± 42	385 ± 36	393 ± 78^∗^
20–40 nm nanospheres	187 ± 5	337 ± 13	373 ± 21	387 ± 76^∗^
50 nm nanorods	288 ± 20	463 ± 48	487 ± 76	496 ± 148^∗^

*^a^Hydrodynamic diameter are expressed as mean ± SD (*n* = 3). ^∗^Statistical analysis was used to compare the significant difference of hydrodynamic diameter between 1 and 96 h (*p* < 0.05).*

### The 96 h Toxicity of AgNPs and AgNO_3_

**FIGURE 2:**
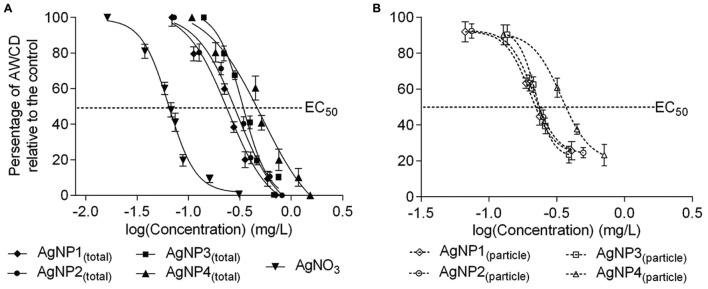
**Dose–response curves of average well color development (AWCD) of soil extracts exposed to suspensions of **(A)** AgNPs_(total)_ and AgNO_3_, and **(B)** AgNPs_(particle)_ expressed as time weighted concentrations.** AWCD are plotted on the *y*-axis, actual log-transformed Ag concentrations are plotted on the *x*-axis. Data are mean ± SD (*n* = 3). (AgNP1:50–80 nm nanoplates, AgNP2:15 nm nanospheres, AgNP3:20–40 nm nanospheres, and AgNP4:50 nm nanorods).

The dose–response curves of AgNPs_(total)_ and AgNO_3_ based on the TWA concentrations are provided in **Figure 2A** As described above, toxicity was expressed as the values of AWCD. It can be seen that all exposures of the microbial community to the nanoparticle suspensions induced significant toxicity. The 50–80 nm nanoplates_(total)_, 15 nm nanospheres_(total)_ and 20–40 nm nanospheres_(total)_ showed much lower 96 h EC_50_ values compared to that of 50 nm nanorods_(total)_. The EC_50_ value derived from the AgNO_3_ curve was 0.064 mg/L, which implied that the Ag^+^ was the most toxic among all the different silver species tested.

The dose–response curves of AgNPs_(particle)_ based on the TWA concentrations are provided in **Figure 2B** These curves could be derived after using the RA model. The 50–80 nm nanoplates_(particle)_, 15 nm nanospheres_(particle)_ and 20–40 nm nanospheres_(particle)_ showed much lower 96 h EC_50_ values of 0.201 mg/L, 0.212 mg/L and 0.222 mg/L, respectively, compared to the 50 nm nanorods_(particle)_ (0.342 mg/L), which indicated that the 50–80 nm nanoplates, 15 nm nanospheres and 20–40 nm nanospheres were much more toxic to soil microbes, compared to the 50 nm nanorods.

The dose–response curves based on initial concentrations of the differently sized and shaped AgNPs on the catabolic capabilities of a microbial community are shown in Supplementary Figure S3. The EC_50_ values of AgNPs_(total)_ and AgNPs_(particle)_ using TWA versus initial concentrations are summarized in **Table 2**

**Table 2:** The EC_50_ values of AgNPs_**(total)**_ and AgNPs_**(particle)**_ expressed on initial concentrations and expressed on time weighted average concentrations.

		EC_50_ (mg/L)^a^		95% Confidence intervals	
		Total	Particle	Total	Particle
Initial concentrations	50–80 nm nanoplates	0.354	0.228	0.331–0.506	0.216–0.242
	15 nm nanospheres	0.409	0.249	0.318–0.394	0.227–0.267
	20–40 nm nanospheres	0.535	0.313	0.487–0.715	0.234–0.264
	50 nm nanorods	0.590	0.402	0.376–0.763	0.361–0.446
TWA^b^	50–80 nm nanoplates	0.242	0.201	0.205–0.286	0.184–0.220
	15 nm nanospheres	0.299	0.212	0.255–0.351	0.198–0.227
	20–40 nm nanospheres	0.337	0.222	0.307–0.369	0.207–0.237
	50 nm nanorods	0.521	0.342	0.380–0.714	0.302–0.387

*^a^EC_*50*_ is half maximal effective concentration. ^b^TWA is time weighted average concentration.*

### Relative Contribution to Toxicity of AgNPs_(particle)_ and AgNPs_(ion)_

The relative contributions of AgNPs_(particle)_ and AgNPs_(ion)_ to the overall toxicity of AgNPs to a microbial community at different effect levels is given in **Table 3** At the EC_20_ level, the contributions of 50–80 nm nanoplates_(particle)_, 15 nm nanospheres_(particle)_, 20–40 nm nanospheres_(particle)_, and 50 nm nanorods_(particle)_ to the overall toxicity were 32–43%. The contribution to toxicity of the particles increased with increasing exposure concentrations. The 50–80 nm nanoplates_(particle)_, 15 nm nanospheres_(particle)_, 20–40 nm nanospheres_(particle)_, and 50 nm nanorods_(particle)_ accounted for about 60–68% of the relative contribution to toxicity at the EC_90_ level of the suspensions.

**Table 3:** Relative contribution of particles and shedding ions of AgNPs to toxicity at different effect levels of nanoparticle suspensions.

		50–80 nm nanoplates		15 nm nanospheres		20–40 nm nanospheres		50 nm nanorods	
		Conc. (mg/L)	Contrib. (%)	Conc. (mg/L)	Contrib. (%)	Conc. (mg/L)	Contrib. (%)	Conc. (mg/L)	Contrib. (%)
EC_20_	Particle	0.067	32	0.075	33	0.137	40	0.176	43
	Ion	0.045	68	0.052	67	0.085	60	0.092	57
EC_ 60_	Particle	0.188	53	0.199	55	0.213	58	0.383	62
	Ion	0.087	47	0.144	45	0.184	42	0.194	38
EC_80_	Particle	0.237	58	0.251	61	0.260	62	0.447	68
	Ion	0.118	42	0.152	39	0.203	38	0.310	32
EC_ 90_	Particle	0.307	60	0.327	63	0.389	64	0.708	68
	Ion	0.182	40	0.193	37	0.209	36	0.467	32

### Community Metabolic Diversity

**FIGURE 3:**
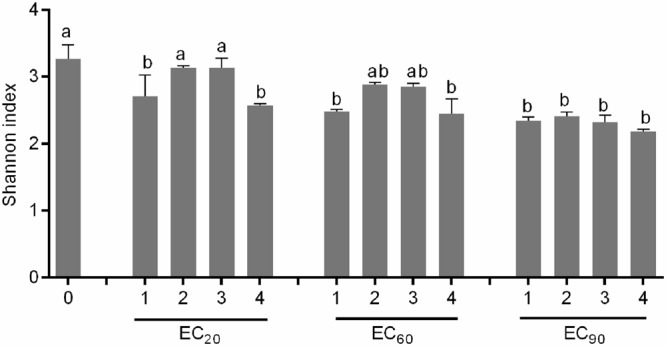
**Functional diversity of microbial communities under the four different AgNPs treatment at the EC_20_, EC_60_, and EC_90_ levels.** The bars are the standard errors of the means (*n* = 3). The different letters above the bar in the same EC level indicate significant differences between different treatments at *p* < 0.05 level (0-control, 1:50–80 nm nanoplates, 2:15 nm nanospheres, 3:20–40 nm nanospheres, and 4:50 nm nanorods).

Toxic effects of AgNPs to the soil microbial community were mainly reflected in a change of the community metabolic diversity. Thus, the functional diversity of microbial communities under the four different AgNPs treatments needed to be further explored. **Figure 3** presents the Shannon index of microbial communities under the four different AgNPs treatments at the EC_20_, EC_60_, and EC_90_ levels. The values of Shannon index decreased with increasing exposure concentrations. No significant difference was observed between the 20–40 nm and 15 nm nanospheres exposure. In contrast, 50–80 nm nanoplates and 50 nm nanorods exposure significantly reduced the community metabolic diversity (*p* < 0.05).

**FIGURE 4:**
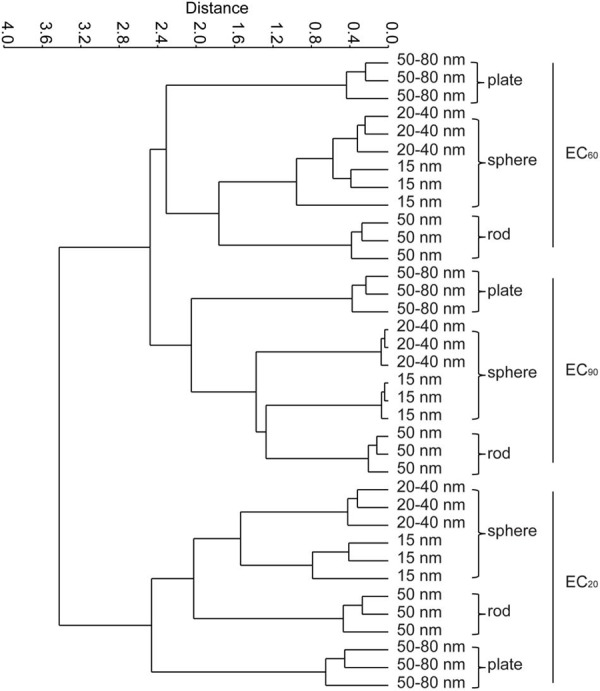
**Classifications of four different AgNPs treatments by cluster analysis according to the metabolic characteristics of the microbial communities at the EC_20_, EC_60_, and EC_90_ levels.** Two-way ANOSIM, Euclidean-based similarity, *n* = 3, EC level *R* = 0.953, *p* = 0.0001; shape *R* = 0.876, *p* = 0.0001, respectively.

Patterns in carbon substrate utilization reflected differences in the functional diversity of the microbial communities among different AgNPs treatments (Supplementary Figure S2 and **Figure 4**). The microbial communities in the control soil extracts were in general able to metabolize 20 of the 31 carbon sources, and the utilization of most of the substrates decreased with the increasing AgNPs concentrations (Supplementary Figure S2). At the EC_20_ level, the utilization of Glycogen was inhibited in all AgNPs exposed microbial communities, however, the ability of microbial communities to utilize Glycyl-L-glutamic acid was stimulated by the AgNPs exposure. At the EC_60_ level, the similar pattern of carbon utilization was only obtained in microbial communities under treatment of 15 and 20–40 nm nanoshperes, which suggested that microbial efficiency in exposure of spherical AgNPs was similar. At the EC_90_ level, exposure to AgNPs significantly inhibited utilization of all substrates (*p* < 0.05).

**Figure 4** shows the cluster analysis of the carbon source metabolisms of the microbial communities under the four AgNPs treatments at the EC_20_, EC_60_, and EC_90_ levels. Microbial metabolic diversity differed significantly between treatments depending on both EC levels and shapes (two-way ANOSIM: EC level *R* = 0.953, *p* < 0.05; shape *R* = 0.876, *p* < 0.05), showing that the developed microbial communities were functionally distinct.

## Discussion

In this study, we found that the dissolution of AgNPs at 1 mg/L increased with time, with 6–13% of the Ag-ion released after 96 h of incubation (**Figure 1**). This was similar to the result obtained by Zhao and Wang (2012) who detected that less than 10% of Ag release from AgNPs at an initial concentration of 1 mg/L. The ion release profiles of NPs was a combined effects affected by the physio-chemical characteristics of NPs, such as particle size, shapes, initial concentrations of NPs and so on (Yang et al., 2014). In our case, the nanoplates AgNPs were demonstrated to be more soluble than the other shapes of AgNPs in the exposure media, and the ion release of nanorods AgNPs were the lowest. Expressing the exposure concentration based on TWA accounts for declining trends in the exposure over time and variability between NP species, and hence gave a more accurate display for exposure concentrations as experienced by microorganisms compared to expressing responses based on the initial concentrations. This decline in exposure concentration also made that the EC_50_ values lower based on TWA than on the initial concentration, and the EC_50_ values of AgNPs_(particle)_ got lumped together for the 50–80 nm nanoplates, 15 nm nanospheres, 20–40 nm nanospheres, and even 50 nm nanorods came more close. Currently, we cannot explain this phenomenon, but it does mean that the toxicity irrespective of the shape is more or less similar, and that the difference within the overall response (expressed as EC_50_ values for AgNPs_(total)_) more likely can be explained by differences in ion shedding from the different shaped particles. Since initial concentrations is currently the most widely used way of expressing the fate of nanoparticles in exposure medium, we incorporated this as well to allow comparison with earlier reported studies. However, based on our observations, we strongly encourage to use TWA concentrations as the TWA approach can capture the condition-related dynamic changes of NPs in exposure medium.

The relative contributions of ions and particles in the effect of NPs on natural microbial communities to date remain uncertain. Previous studies have focused more on the toxicity of AgNPs suspensions. Topuz and van Gestel (2015) concluded that the toxicity of citrate-coated AgNPs and polyvinylpyrrolidone-coated AgNPs on *Enchytraeus crypticus* probably was mainly attributable to AgNPs_(ion)_. Our results showed that at lower exposure concentrations (EC_20_ level), the contribution of the AgNPs_(ion)_ was higher than those of AgNPs_(particle)_, while the contribution to toxicity of the AgNPs_(particle)_ increased with increasing exposure concentrations. At the EC_90_ level, AgNPs_(particle)_ dominated the toxicity. Similar findings were reported for AgNPs by Wang et al. (2012). The higher contribution to toxicity by AgNPs_(particle)_ could be caused by the existence of a particle-mediated mechanism. The AgNPs_(particle)_ could also enter the cell to generate reactive oxygen species, leading to damage to proteins and nucleic acids inside the bacterial cell, and finally inhibition of cell proliferation (Mcshan et al., 2014). Our study did not account for naturally relevant complexity (e.g., pH, temperature) nor compounds that can either synergistically or antagonistically affect the toxicity of AgNPs (e.g., biofilms, humic acids; Ikuma et al., 2015), but clearly hints that contributions of ions and particles in the adverse effects of NPs pollution on microbial communities largely depend on exposure levels in the environment.

The reactivity and bactericidal properties of AgNPs has been observed to change for differently sized and shaped particles (Pal et al., 2007; Sillen et al., 2015). Our results clearly showed that different shaped AgNPs severely disrupted metabolic processes of a natural soil microbial community. Studies have shown that AgNPs toxicity was size dependent at low concentrations. Ivask et al. (2014) observed that AgNPs of 1–10 nm were preferentially bound to cell membranes and were incorporated into bacteria, whereas larger AgNPs were not. In the present study, the 50 nm nanorods were found to have an EC_50_ level about two times higher than that of the 20–40 nm nanospheres and the 15 nm nanospheres. It showed that soil microbes were more vulnerable to smaller sized AgNPs than to larger sized ones. In addition, the surface area of nanosilver shapes is a key factor for controlling antimicrobial activity. The effect of AgNPs shape had also been observed in bacteria, with nanoplates showing a higher degree of toxicity (Pal et al., 2007). Sadeghi et al. (2012) also found the nanoplates solutions had a good anti-bacterial activity for both *Staphylococcus aureus* and *Escherichia coli* compared with nanorods and nanospheres. In our results, 50–80 nm nanoplates were found to be more toxic than the 50 nm nanorods. Although size was not fully represented across all our treatments and therefore prevented inference on the role of size in AgNP toxicity, these data seemed to suggest that AgNP toxicity was also size dependent. The efficient antimicrobial activity of the Ag nanoplates was ascribed to their sharp corners and edges and large areas of active crystal plane, which led to the higher amount of leaching Ag-ion (Lu et al., 2015).

Although AgNPs are well known for their anti-microbial properties, their effect on the functional diversity of natural communities remains less well understood. Our study showed that, while the overall microbial diversity and activity was comparable among treatments, the functional diversity was clearly altered depending on both concentration and shape of AgNPs. Although the use of Biolog Ecoplates did not allow firm conclusions on the actual mechanisms underlying shifts in functional diversity, it could be speculated that AgNPs alter the microbial community by adversely affecting specific enzymes and utilization of specific substrates. For instance, utilization of glycogen was inhibited in the presence of even low AgNP concentrations. Several enzymes taking part in glycogen metabolism responded to metabolites that signaled the energy need of the bacterial cell (Berg et al., 2012). Hence, the addition of AgNPs caused damage to the activation of enzymes, especially to the ones that could metabolize Glycogen. Subsequently, at higher exposure concentrations of AgNPs, the particles caused damage to the functioning of microbial communities, which likely cascaded towards distorted ecosystem processes (Kumar et al., 2011). Irrespective of the mechanism underlying our observation, AgNPs can thus clearly affect the functional composition of soil microbial communities. Disentangling naturally relevant risks, biochemical interactions and AgNPs effects on bacteria-mediated processes are thus promising areas of future research.

## Conclusion

Our results clearly showed that the relative contribution of AgNPs_(particle)_ could increase with increasing exposure concentrations and that AgNPs shape could differentially affect the functional composition of soil microbial communities. The main release routes of NPs to the environment is via wastewater eﬄuents and deposition (McKee and Filser, 2016). As residence times of NPs in soils and sediments in general exceed typical residence times of NPs in aquatic systems, both soils and sediments can be considered the ultimate sinks of NPs in which concentrations of non-degradable or slowly degradable NPs will gradually built up. Although we used moist soil for our laboratory incubations, we thus expect that the outcome of this study also applies to aquatic sediments as AgNPs typically agglomerate in aqueous environments and thereby precipitate onto the surface of both soil and sediment particles due to the high density of Ag (e.g., Bradford et al., 2009). However, these results were obtained in simplified systems under laboratory conditions and did not include ecologically relevant compounds that potentially affect the toxicity of AgNPs. Therefore, our results should be interpreted cautiously as it remains uncertain whether patterns observed in the presently studied laboratory incubations reflected those occurring in natural systems. Not accounting for complexity likely resulted in an overestimated toxicity in our setup, yet the emerging pattern that relative contributions of ions and particles can shift depending on exposure concentrations likely resonates with relative contributions of ions and particles in natural environments. This outcome thus hints that both exposure concentrations and shape of AgNPs_(particle)_ can play a significant role in the functional composition of microbial communities and in turn their associated ecosystem processes, warranting consideration of the effect of NPs on microbial functioning in environmental science and management.

## Author Contributions

YZ conceived the study, performed the experiments, analyzed the data and drafted the manuscript. EH helped the interpretation of data. MW helped the Biology experiments. EH, WP and MV helped the critical revision of manuscript. All authors contributed to improve the manuscript and approved the final version of the manuscript.

## Conflict of Interest Statement

The authors declare that the research was conducted in the absence of any commercial or financial relationships that could be construed as a potential conflict of interest.
