# Supplementary material for: Silver Nanoparticles, Ions, and Shape Governing Soil Microbial Functional Diversity: Nano Shapes Micro
Source: Front Microbiol. 2016 Jul 25;7:1123. doi: 10.3389/fmicb.2016.01123 (PMC4959451; doi:10.3389/fmicb.2016.01123)
Supplement: Supplementary file 1 [file Data_Sheet_1.DOCX]

***Supplementary Material***

**Nano shapes micro: Silver nanoparticles, ions and shape governing soil microbial functional diversity**

**Yujia Zhai^1^*, Marja Wouters^2^, Ellard R. Hunting^1^, Willie J.G.M. Peijnenburg^1,2^, and Martina G. Vijver^1^**

*** Correspondence:** Yujia Zhai: y.zhai@cml.leidenunv.nl

1. **Supplementary Figure** **and Tables**
2. **Supplementary Figure**

**
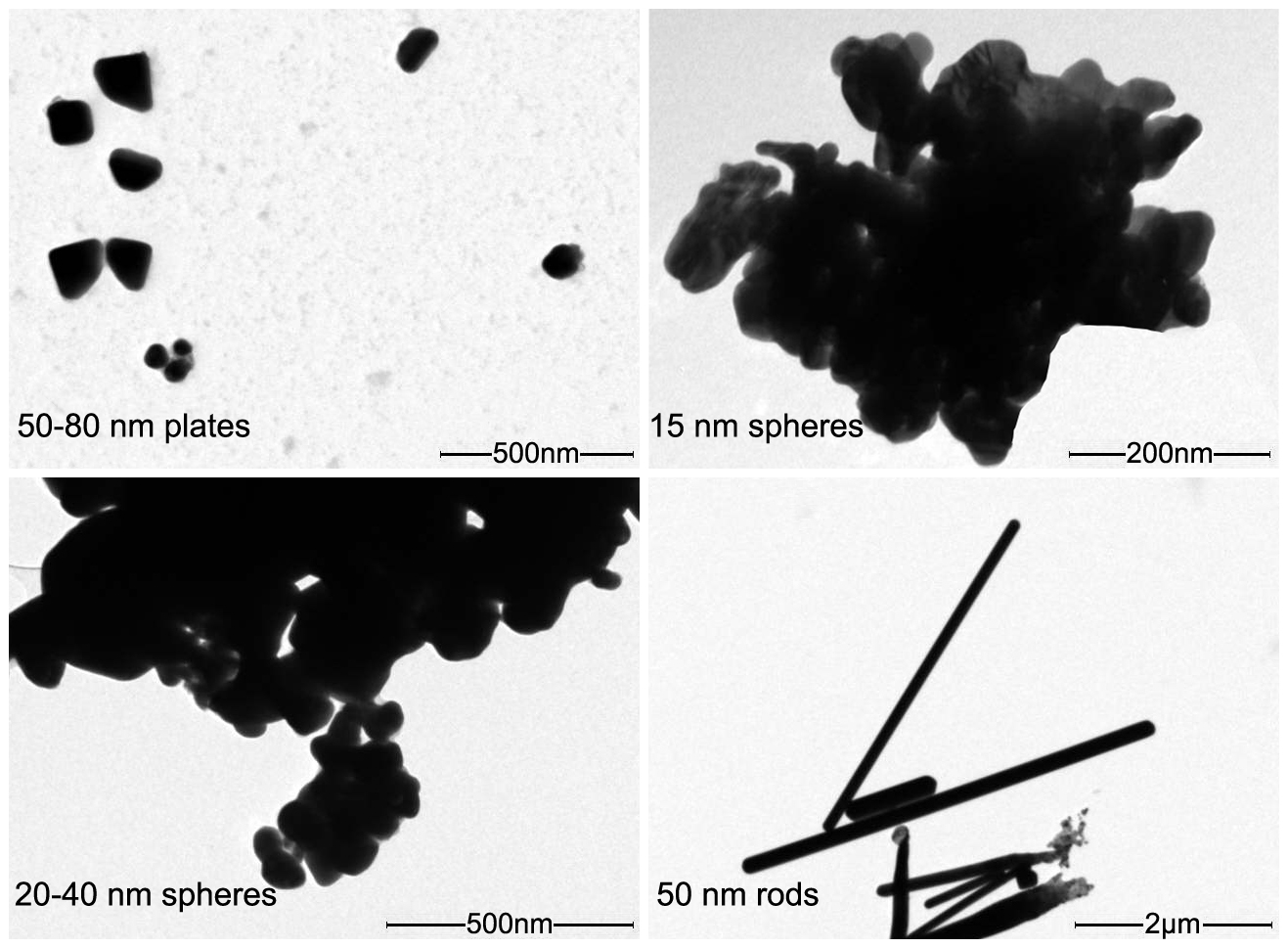
**

**Supplementary Figure 1. Transmission electron microscopic micrographs of the silver nanoparticles used in this study (50-80 nm plates, 15 nm spheres, 20-40 nm spheres and 50 nm rods particles) dissolved in egg water sensu Hua et al., 2014.**


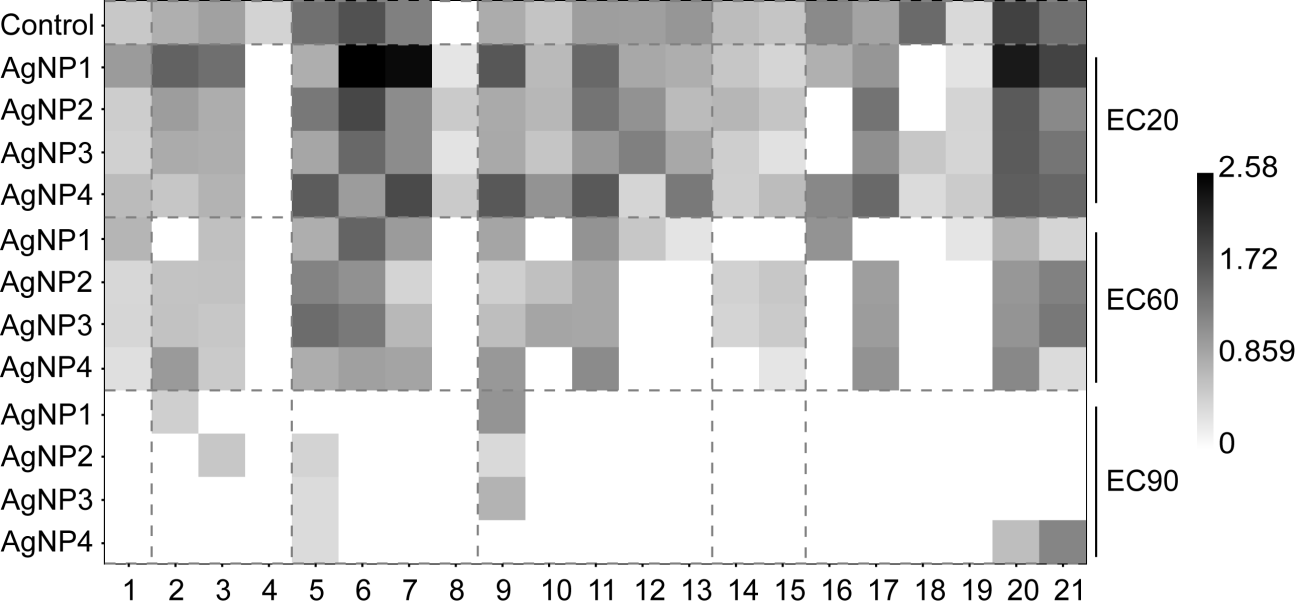


**Supplementary Figure 2. A heatmap for demonstrating utilization variation of substrates under four different AgNPs treatments at the EC20, 60 and 90 levels.** (1-Putrescine, 2-Tween 80, 3-Tween 40, 4- Glycogen, 5- L-Asparagine, 6-L-Serine, 7- Glycyl-L-glutamic acid, 8-L-Arginine, 9-D-Glucosaminic acid, 10-D-Galacturonic acid, 11-D-Galactonic acid γ-Lactone, 12-4-Hydroxy benzoic acid, 13-Itaconic acid, 14-Glucose -l-phosphate, 15-Pyruvic acid methyl ester, 16-D-Mannitol, 17-N-Acetyl-D-glucosamine, 18-β-Methyl-D-glucoside, 19-D-Cellobiose, 20-D-Xylose, 21-i-Erythritol; AgNP1-50-80 nm nanoplates, AgNP2-20-40 nm nanospheres, AgNP3-15 nm nanospheres, AgNP4-50 nm nanorods).


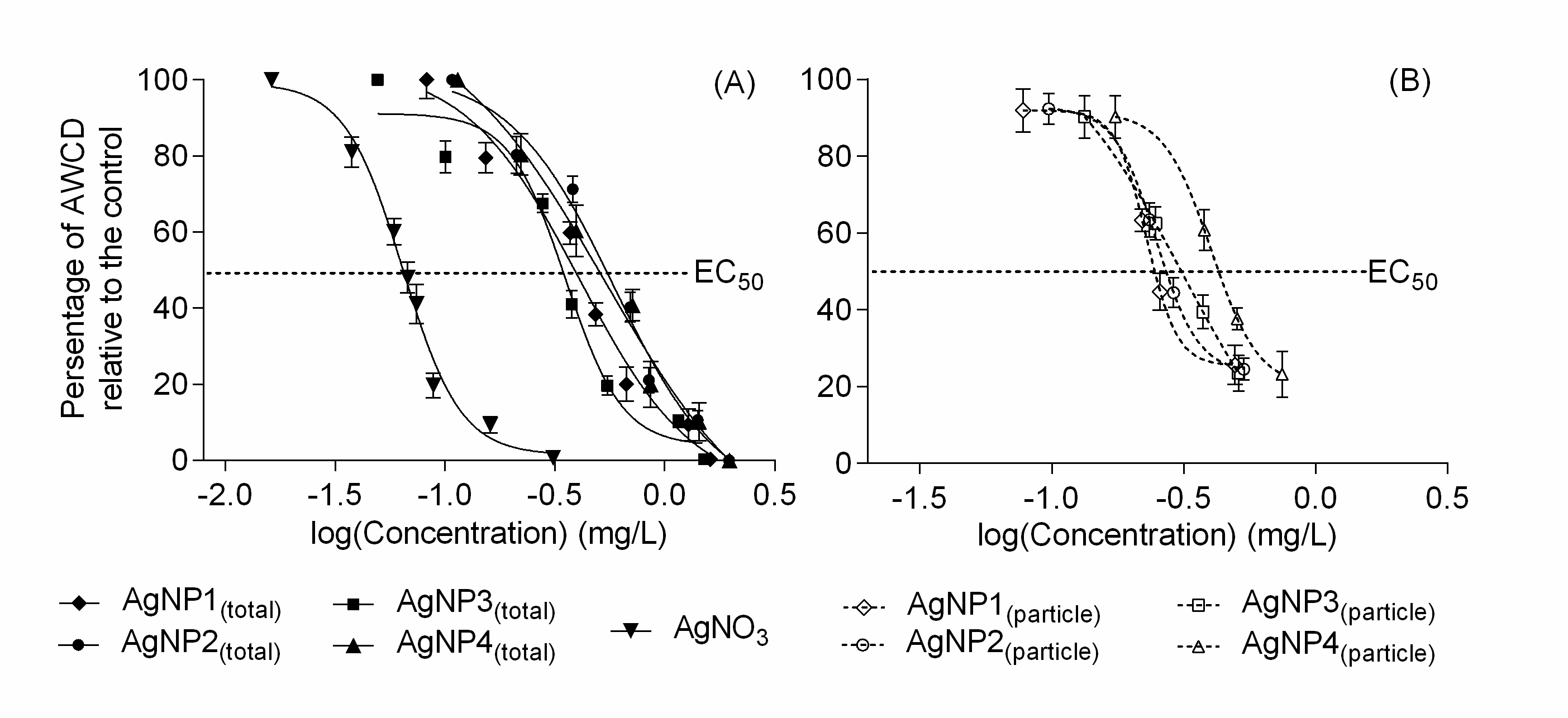


**Supplementary Figure 3. Dose-response curves of AWCD of soil extracts exposed to suspensions of (A) AgNPs_(total)_ and AgNO_3_, and (B) AgNPs_(particle)_ expressed as initial concentrations.** AWCD are plotted on the y-axis, actual log-transformed Ag concentrations are plotted on the x-axis. Data are mean ± SD (n= 3). (AgNP1-50-80 nm nanoplates, AgNP2-15 nm nanospheres, AgNP3-20-40 nm nanospheres, AgNP4-50 nm nanorods).**Supplementary Table**

**Supplementary Table 1.** Individual carbon sources and their classification in Biolog EcoPlate.

| Carbon source | Substrate | Chemical formula | Serial No. |
| --- | --- | --- | --- |
| Amines/amides | G4: Phenyethylamine | C_8_H_11_N | 30 |
|  | H4: Putrescine | C_4_H_12_N_2_ | 31 |
| Amino acids | A4: L-Arginine | C_6_H_14_N_4_O_2_ | 24 |
|  | B4: L-Asparagine | C_4_H_8_N_2_O_8_ | 25 |
|  | C4: L-Phenylalanine | C_9_H_11_NO_2_ | 26 |
|  | D4: L-Serine | C_3_H_7_NO_3_ | 27 |
|  | E4: L-Threonine | C_4_H_9_NO_3_ | 28 |
|  | F4: Glycyl-L-glutamic acid | C_7_H_12_N_2_O_5_ | 29 |
| Carbohydrates | A2: β-Methyl-D-glucoside | C_7_H_14_O_6_ | 8 |
|  | B2: D-Xylose | C_5_H_10_O_5_ | 9 |
|  | C2: i-Erythritol | C_4_H_10_O_4_ | 10 |
|  | D2: D-Mannitol | C_6_H_14_O_6_ | 11 |
|  | E2: N-Acetyl-D-glucosamine | C_8_H_15_NO_6_ | 12 |
|  | G1: D-Cellobiose | C_12_H_22_O_11_ | 6 |
|  | H1: α-D-Lactose | C_12_H_22_O_11_ | 7 |
| Carboxylic acids | A3: D-Galactonic acid γ-Lactone | C_6_H_10_O_6_ | 16 |
|  | B3: D-Galacturonic acid | C_6_H_10_O_7_ | 17 |
|  | C3: 2-Hydroxy benzoic acid | C_7_H_6_O_3_ | 18 |
|  | D3: 4-Hydroxy benzoic acid | C_7_H_6_O_3_ | 19 |
|  | E3: γ-Hydroxy butyric acid | C_4_H_8_O_3_ | 20 |
|  | F2: D-Glucosaminic acid | C_6_H_13_NO_6_ | 13 |
|  | F3: Itaconic acid | C_5_H_6_O_4_ | 21 |
|  | G3: α-Keto butyric acid | C_4_H_6_O_3_ | 22 |
|  | H3: D-Malic acid | C_4_H_6_O_5_ | 23 |
| Miscellaneous | B1: Pyruvic acid methyl ester | C_4_H_6_O_3_ | 1 |
|  | G2: D-Glucosaminic acid | C_6_H_13_O_9_P | 14 |
|  | H2: D,L-α-Glycerol-phosphate | C_3_H_9_O_6_P | 15 |
| Polymers | E1: α-Cyclodextrin | C_36_H_60_O_30_ | 4 |
|  | F1: Glycogen | (C_6_H_10_O_5_)*_n_* | 5 |
|  | C1: Tween 40 | - | 2 |
|  | D1: Tween 80 | - | 3 |

**Supplementary Table 2.** Statistics of the nonlinear fit of ion release profile for each AgNPs_(total)_ and AgNPs_(particle)_.

|  |  | | Total |  |  | Particle |  |
| --- | --- | --- | --- | --- | --- | --- | --- |
|  | *K* | | *P* | *R*^2^ | *K* | *P* | *R*^2^ |
| 50-80 nm nanoplates | | 0.019 | 0.501 | 0.839 | 0.020 | 0.423 | 0.850 |
| 15 nm nanospheres | | 0.015 | 0.484 | 0.942 | 0.014 | 0.406 | 0.916 |
| 20-40 nm nanospheres | | 0.010 | 0.396 | 0.811 | 0.009 | 0.314 | 0.842 |
| 50 nm nanorods | | 0.025 | 0.618 | 0.717 | 0.023 | 0.583 | 0.744 |
